# Acute Exposure to Fluoxetine Alters Aggressive Behavior of Zebrafish and Expression of Genes Involved in Serotonergic System Regulation

**DOI:** 10.3389/fnins.2017.00223

**Published:** 2017-04-25

**Authors:** Antonia Theodoridi, Aleka Tsalafouta, Michail Pavlidis

**Affiliations:** Laboratory of Fish Physiology, Department of Biology, University of CreteHeraklion, Greece

**Keywords:** zebrafish, fluoxetine, social stress, aggression, serotonin

## Abstract

Zebrafish, *Danio rerio*, is an emerging model organism in stress and neurobehavioral studies. In nature, the species forms shoals, yet when kept in pairs it exhibits an agonistic and anxiety-like behavior that leads to the establishment of dominant-subordinate relationships. Fluoxetine, a selective serotonin reuptake inhibitor, is used as an anxiolytic tool to alter aggressive behavior in several vertebrates and as an antidepressant drug in humans. Pairs of male zebrafish were held overnight to develop dominant—subordinate behavior, either treated or non-treated for 2 h with fluoxetine (5 mg L^−1^), and allowed to interact once more for 1 h. Behavior was recorded both prior and after fluoxetine administration. At the end of the experiment, trunk and brain samples were also taken for cortisol determination and mRNA expression studies, respectively. Fluoxetine treatment significantly affected zebrafish behavior and the expression levels of several genes, by decreasing offensive aggression in dominants and by eliminating freezing in the subordinates. There was no statistically significant difference in whole-trunk cortisol concentrations between dominant and subordinate fish, while fluoxetine treatment resulted in higher (*P* = 0.004) cortisol concentrations in both groups. There were statistically significant differences between dominant and subordinate fish in brain mRNA expression levels of genes involved in stress axis (*gr, mr*), neural activity (*bdnf*, *c-fos*), and the serotonergic system (*htr2b, slc6a4b*). The significant decrease in the offensive and defensive aggression following fluoxetine treatment was concomitant with a reversed pattern in *c-fos* expression levels. Overall, an acute administration of a selective serotonin reuptake inhibitor alters aggressive behavior in male zebrafish in association with changes in the neuroendocrine mediators of coping styles.

## Introduction

Aggressive and defensive behavior finds its deep source in evolution and Darwinian fitness, since it is of essential adaptive nature (Oliveira et al., 2011). Aggressive behaviors are widely observed in a variety of animal species, especially those living in groups (Drews, 1993) and they are considered a way to form and maintain social hierarchies (Paull et al., 2010). Fish display aggressiveness in an effort to gain access to environmental resources, mates and territories, and to protect their offspring (Huntingford and Turner, 1987).

Zebrafish, *Danio rerio*, in nature forms shoals, yet when kept in pairs it exhibits an aggressive, agonistic, and anxiety-like behavior that most of the time leads to the formation of hierarchies. Hierarchies consist of a dominant-subordinate relationship and have been observed in male (Larson et al., 2006; Pavlidis et al., 2011) as well as female zebrafish (Dahlbom et al., 2011). More precisely, dominant males exhibit aggression by chasing and occasionally biting the subordinate males, thus gaining exclusive access to the upper two thirds of the experimental tank. Although hierarchies formed are stable, it has been shown that social status can be reversed following various environmental or husbandry conditions. In particular, treatment with antidepressant substances temporarily reversed dominant social status in lizards (Deckel, 1996), while in crustaceans following exposure to artificial serotonin subordinates turned into aggressive dominant males (Huber et al., 1997).

Serotonin (5-HT) has been found to play a significant regulatory role on stress (McKittrick et al., 1995; Emerson et al., 2000), social interactions (Kravitz, 2000), and aggression (Raleigh et al., 1991; Huber et al., 1997) in a wide variety of species. It is indicated that serotonergic activity acts on aggression by repressing it (Popova, 2008), as they are inversely correlated (Ferris et al., 1997; Deckel et al., 1998). While scientific evidence suggests that the serotonergic system is connected to the establishment and maintenance of social rank, it is still unclear whether social rank itself is the cause or the consequence of the differences observed at the physiology and the behavior between dominant and subordinate individuals (Øverli et al., 2004).

Fluoxetine is a selective serotonin re-uptake inhibitor (SSRI; Wong et al., 1995), a compound used widely as an antidepressant toward the treatment of depression, anxiety and personality disorders (Rossi et al., 2004). Its mechanism relies on the inhibition of the reuptake of serotonin into the presynaptic cell, thus increasing its extracellular level (Vaswani et al., 2003). Serotonin is an important neurotransmitter linked frequently to depressive conditions (Albert and Benkelfat, 2013; Blier and El Mansari, 2013). Wide international research implicated serotonin on mood regulation and learning (Meneses and Liy-Salmeron, 2012), appetite (Lam et al., 2011), sleep (Portas et al., 2000), memory (Seyedabadi et al., 2014).

Aggression is used commonly by dominant individuals to enable them to occupy territories over spawning sites and protect their status from their subordinates, therefore there is a close association between aggression and social rank (Filby et al., 2010). Social rank and dominance hierarchies constitute a form of stress, characterized as social stress. As it has been shown that chronic administration of fluoxetine has an anxiolytic effect on zebrafish (Dulawa et al., 2004; Egan et al., 2009; Wong et al., 2013), the drug could be used as a neuropharmacological tool to modify social stress experienced by zebrafish. The purpose of this study was to investigate associated changes in molecular and endocrine modulators involved in the regulation of stress and aggressive behavior in male zebrafish. The genes that were quantified, were selected due to their role in the HPI axis activation (*gr, mr, hcrt, avt, prl*), the serotonergic system (*htr1Aa, htr1b, htr2b, slc6a4a, slc6a4b*) and neural activity (*c-fos, bdnf*).

## Materials and methods

### Animal and husbandry conditions

Wild-type zebrafish (*Danio rerio*) of Malaysian origin were obtained from a Greek wholesaler and maintained in 2 × 250-L aquaria (holding tanks) at the installations of the Fish Physiology Laboratory, University of Crete. Aquaria were equipped with a biological filter (Eheim external canister filter; EHEIM GmbH & Co. KG) and facilities for temperature and photoperiod control as well as for aeration and oxygen supply. Water temperature was set at 26°C and photoperiod at 12L:12D. Water chemical parameters were monitored on a daily (dissolved oxygen, pH) or weekly base (ammonia, nitrite and nitrate). Fish were fed daily with industrial aliment (Principal Food, Sera Vipan).

### Experimental design

Male zebrafish were anesthetized in MS222, weight and length measurements were conducted and a tiny part of the upper or lower caudal fin lobe was cut for identification. The experiment was conducted in two phases. In Phase I, fish that did not differ in length more than 1 mm were paired and transported to 2 L glass aquaria, where they were left to interact overnight. Each tank was then recorded for 5 min using a digital video camera. Only the pairs that showed a stable and strong dominant-subordinate relationship were used for the Phase II of the experiment. Dominant-subordinate relationships that did not reverse throughout the experiment constituted stable and strong hierarchies.

In Phase II, two different groups of pairs (each one consisted of a dominant and subordinate individual) were formed; the fluoxetine treated (*n* = 8 pairs) and the non-treated (*n* = 8 pairs). More specifically, each pair of the fluoxetine treated group was put in 200 ml rectangular food storage containers with a dose of 5 mg L^−1^ fluoxetine (Sigma F-132, Sigma-Aldrich) while each pair of the non-treated group was placed in 200 ml containers with plain tank water. In all pairs, the dominant and subordinate fish were separated by a small black net placed within the container. After 2 h of exposure, pairs were transported to the initial 2 L tanks. According to the literature (Blaser and Gerlai, 2006), maximum dose of fluoxetine administered to adult zebrafish is often given at concentrations up to 0.1 mg L^−1^, but administered chronically over a period of 2-weeks. The high dose of fluoxetine used in the present experiment was based on previous data published in mice and rats (5–20 mg kg^−1^; Blanchard et al., 1997; Conley and Hutson, 2007), piauçu fish, *Leporinus macrocephalus* (10 mg kg^−1^; Barbosa et al., 2012), *Betta splendens* (Kohlert et al., 2012), and zebrafish (2.5 or 3 mg kg^−1^; Norton et al., 2011; Maximino et al., 2013, 2014), showing a clear effect on behavior.

Pair's behavior was observed for an hour, recorded and then fish were netted and immediately sacrificed by immersion in ice-cold water. Brains were dissected and samples from two fish of the same coping style were pooled, frozen in liquid nitrogen and placed at −80°C for mRNA expression analysis. Then, fish head and caudal fin were cut and trunks were weighed and frozen for cortisol determination.

### Ethics statement

The experiments were approved by the Departmental Animal Care Committee following the 3Rs principle, and they were implemented in accordance with the Greek (PD 56/2013) and EU (Directive 63/2010) legislation on the care and use of experimental animals. The Animal House facilities at the Department of Biology, University of Crete, are certified by the Veterinary Unit of the Region of Crete for the rearing (EC91-BIObr-09) and use (EL91-BIOexp-10) of laboratory animals for scientific purposes.

### Behavioral analysis

Behavioral data were obtained using VideoLAN—VLC media player with optional slow-motion analysis. Dominant and subordinate behavior was quantified according to specific parameters. The duration, proportionate to whole session, of chasing and the number of attacks were associated to dominant behavior, while the duration, relative to session, of freezing was associated to the subordinate behavior. Chasing, according to Paull et al. (2010), was identified as “direct/aggressive swim toward another fish in the aquarium causing to increase its speed and possibly change direction.” An attack of the dominant to the subordinate was set as an attack that may or may not lead to biting trials (Pavlidis et al., 2011). Freezing according to Blaser and Gerlai (2006), was stated as “a motionless state during which only the gills and, occasionally the eyes may move.” Freezing incidents were observed mainly when the subordinate fish was restricted by the dominant fish in the lower one third of the tank and mostly in a corner, and in some rare cases right below the water surface.

### Cortisol determination

Cortisol extraction was performed according to de Jesus et al. (1991) and Pavlidis et al. (2011). Briefly, trunk samples were partially thawed on ice and homogenized in 5 × (w/v), ice-cold, phosphate-buffered saline (pH 7.4) with a rotor homogenizer. Cortisol was extracted from 2 × 250 μL of homogenate with 3 ml of diethyl ether. The water phase of the extract was allowed to freeze by placing tubes in −80°C and the combined diethyl ether layer was transferred into a new tube. The ether was evaporated by placement of tubes in a 45°C water bath for 1 h and in room temperature for an additional 3 h. Samples were then reconstituted in 250 μl of an enzyme immunoassay buffer. Cortisol was quantified by the use of a commercial enzyme immunoassay kit (Cayman Chemical, MI, USA) previously evaluated (Pavlidis et al., 2011). As previously evaluated, the recovery of cortisol was 91.2 ± 4.3% (mean ± SEM, *n* = 2). All samples were tested in duplicate.

### RNA isolation and cDNA synthesis

Whole brain samples were disrupted and homogenized in 600 μl RLT plus buffer (RNeasy Plus Mini Kit Qiagen, Valencia, USA) using the TissueRuptor (Qiagen, Hilden, Germany). Total RNA was extracted using RNeasyPlus Mini Kit (Qiagen Inc., CA, USA). Measurement of the absorbance at 260 and 280 nm using the Nanodrop® ND-1000 UV–Vis spectrophotometer (Peqlab, Erlangen, Germany) was conducted in order to determine RNA yield and purity. The integrity of RNA was tested by electrophoresis in 1% agarose gels. Additionally, no band of genomic DNA was observed. One microgram of total RNA was reverse-transcripted using QuantiTect Reverse Transcription kit (Qiagen Inc., CA, USA) according to the instructions of the manufacturer.

### Quantitative real-time PCR (qPCR)

The mRNA expression of genes encoding for glucocorticoid receptor (*gr*), mineralocorticoid receptor (*mr*), *c-fos*, hypocretin/orexin (*hcrt*), brain-derived neurotrophic factor (*bdnf*), arginine vasotocin (*avt*), prolactin (*prl*), 5-hydroxytryptamine (serotonin) receptor 1Aa (*htr1Aa*), 1B (*htr1b*), and 2B (*htr2b*), solute carrier family 6 (neurotransmitter transporter), member 4a (*slc6a4a*), and 4b (*slc6a4b*; primers, Table 1) was determined in brain samples of non-treated and fluoxetine treated fish with quantitative polymerase chain reaction (qPCR) assays. Quantitative real-time PCR was performed on the CFX Connect™ Real-Time PCR Detection System (Bio-Rad) using the KAPA SYBR^R^ FAST qPCR Kits (KAPA Biosystems, USA). Cycling parameters were as follows: 95°C for 3 min (HotStarTaq DNA Polymerase activation step) followed by 36 cycles at 95°C for 20 s (denaturation step) and 60°C for 30 s (annealing step). Fluorescence changes were monitored with SYBR Green after every cycle. Dissociation curve analysis was performed at the end of the cycles to ensure that single amplifications were obtained. A standard curve was constructed for each gene, using four serial dilutions (1:5) of a pool of all cDNA samples by graphing the negative log of the dilution factor against the relative cycle threshold value. To be considered suitable for analysis, each primer pair was required to have a linear standard curve with an r2 value above 0.98 and primer amplification efficiency between 95 and 105%. The stability of *b-actin* was validated and proved to be a suitable reference gene that served as internal control. Results were evaluated with the Bio-Rad CFX Manager 2.1 software. The data were calculated by the comparative method using C_t_ values of β*-actin* as the reference control.

**Table 1:** **Primers design**.

**Gene**	**Forward primer**	**Reverse primer**
*b-actin*	5′ TGTCCCTGTATGCCTCTGGT 3′	5′ AAGTCCAGACGGAGGATGG 3′
*Gr*	5′ ACAGCTTCTTCCAGCCTCAG 3′	5′ CCGGTGTTCTCCTGTTTGAT 3′
*Mr*	5′ CCCATTGAGGACCAAATCAC 3′	5′ AGTAGAGCATTTGGGCGTTG 3′
*c-fos*	5′ TGAAACTGACCAGCTTGAGGAT 3′	5′ GTGTGCGGCGAGGATGAA 3′
*Bdnf*	5′ GGCGAAGAGCGGACGAATATC 3′	5′ AAGGAGACCATTCAGCAGGACAG 3′
*Avt*	5′ TCGTCTGCCTGCTACATCCA 3′	5′ TCCGGCTGGGATCTCTTG 3′
*Prl*	5′ GCTCGGTCTCTGCTGTTG 3′	5′ GGTGTTGCGTTCTGGATGT 3′
*Htr5-1Aa*	5′ CAGAGCAGAGCAGCACAAG 3′	5′ TGGTCTGAGAGTTCTGGTCTAATC 3′
*Htr5-1b*	5′ GTGTCGGTGCTCGTGATG 3′	5′ CAGCCAGATGTCGCAGATG 3′
*Htr5-2b*	5′ GCTGCTCATTCTTCTGGTCAT 3′	5′ GTTAGTGGCGTTCTGGAGTT 3′
*slc6a4a*	5′ GTCTCCAATGGTTATCGCAGTA 3′	5′ GATGACCGACAACAGGAAGT 3′
*slc6a4b*	5′ GAATCCTCTGGGCTTGGTAATG 3′	5′ GCTGAAGTAGACAATGGTGAAGAT 3′
*Orexin*	5′ TCTACGAGATGCTGTGCCGAG 3′	5′ CGTTTGCCAAGAGTGAGAATC 3′

### Statistical analyses

All statistical analyses were performed with SigmaStat v3.1 software (Jandel Scientific). All data are presented as means ± standard error of the mean (S.E.M.). Data were initially screened for normality and homogeneity and if needed, they were log transformed. The analysis of behavioral data was performed using two-way ANOVA. Statistical comparisons of trunk cortisol concentration between the coping styles and treatment groups were made using two-way ANOVA. Two-way ANOVA was also used to determine significant differences in the relative mRNA expression levels of candidate genes between the coping styles and treatment groups. If significant (*P* < 0.05), Holm-Sidak comparison test was applied to identify groups that were significantly different. Graphs were generated using GraphPad Prism software (USA).

## Results

### Behavioral data

**Figure 1:**
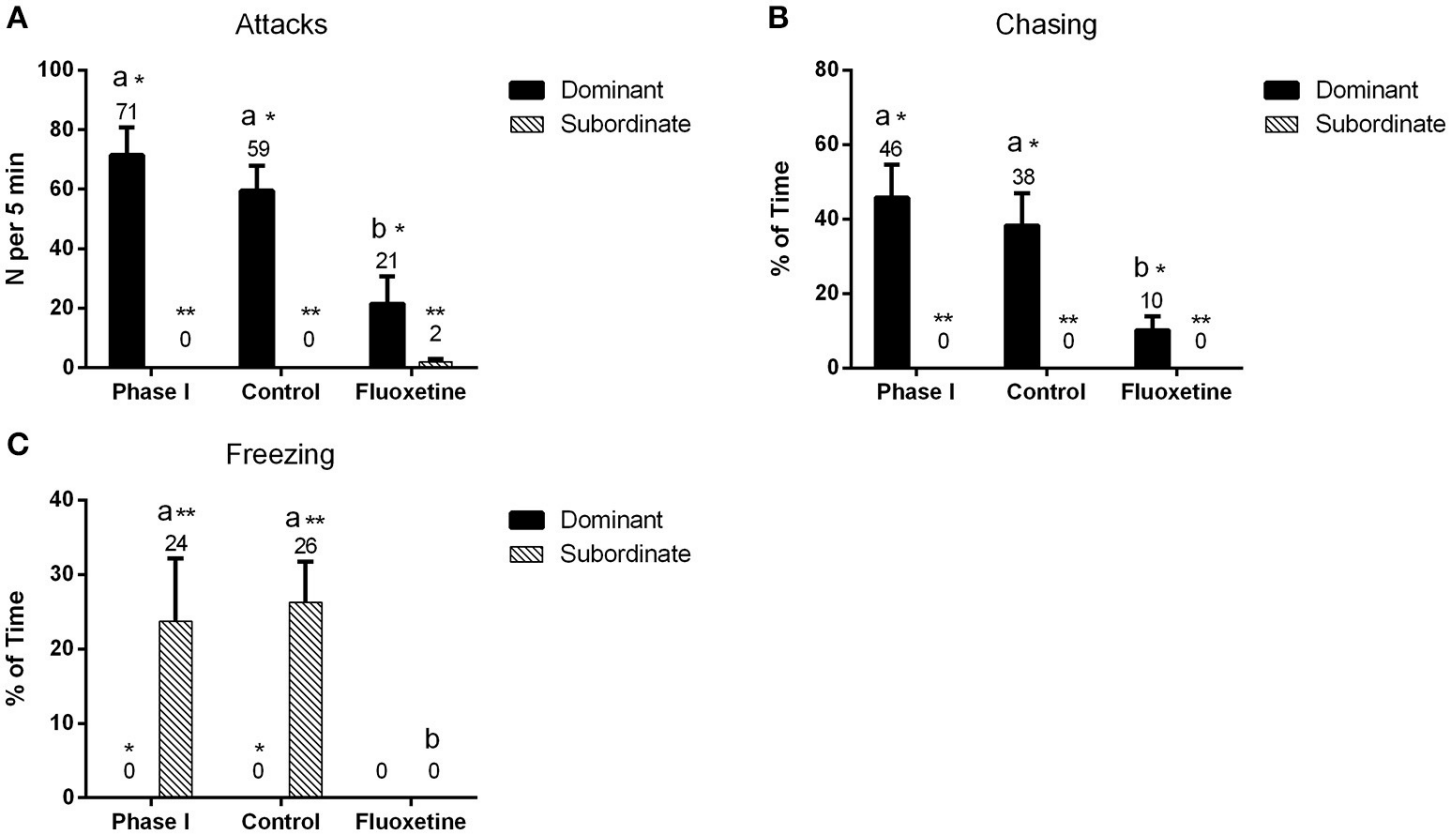
**Quantification of dominant and subordinate behavior throughout the course of the experiment**. “Phase I” refers to the part of the experiment prior to any treatment, “control” represents the group of individuals that did not receive any fluoxetine and “fluoxetine” the group that received a fluoxetine treatment. Attacks **(A)** indicate the number of attacks per session (5 min) and chasing **(B)** the amount of time relative to session spent by the fish in chasing its conspecific. Freezing **(C)** was quantified by measuring the amount of time relative to session spent by the fish on freezing. Bars indicate mean values of the observed time (%) or the number of incidents (*N*) ± S.E.M. (*n* = 8). Mean values are listed above the bar graphs. Letters indicate statistically significant differences (*P* < 0.05) among the three experimental groups, while asterisks differences among the coping styles.

Fluoxetine treatment significantly affected the behavior of zebrafish pairs (Figure 1). Dominants treated with fluoxetine displayed a decrease in the aggressive behavior, expressed through a statistically significant decrease in the number of attacks (*P* < 0.001) as well as a statistically significant decrease in the amount of time (relative to session) spent chasing the subordinates (*P* < 0.001), compared to their behavior in phase I. On the contrary, dominants that did not receive any fluoxetine (control group) did not exhibit any statistically significant difference compared to the behavior observed during phase I. Subordinates treated with fluoxetine, in all pairs, did not show any sign of freezing after fluoxetine treatment (*P* = 0.004); on the contrary they showed some minor incidents of attacks toward the dominants. Additionally, subordinates in the control group showed no decrease in their freezing behavior and no incidents of attacks toward the dominants.

### Cortisol

**Figure 2:**
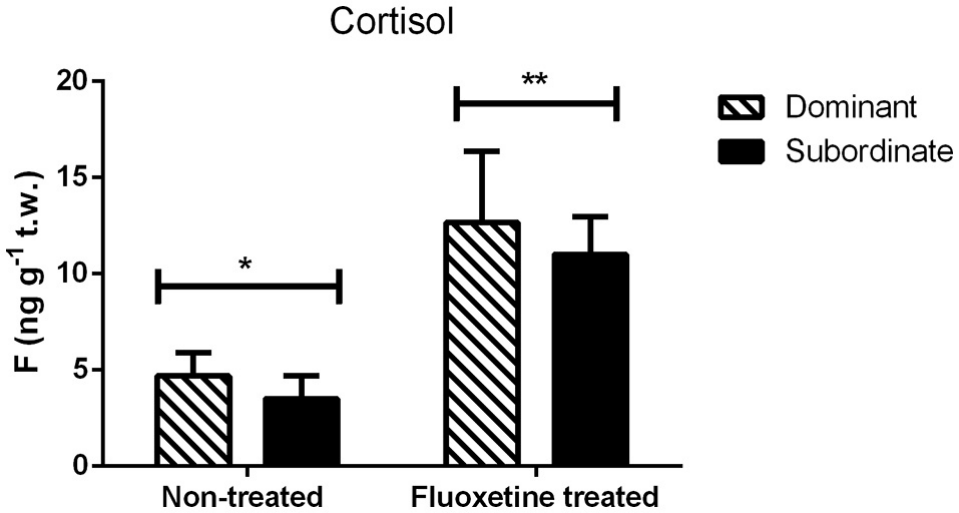
**Trunk cortisol (F) concentrations (mean ± S.E.M., *n* = 8 per group) in non-treated and fluoxetine treated pairs**. Statistical analysis performed using two-way ANOVA. Different asterisks indicate statistically significant differences among the two treatments (fluoxetine or non-treated groups).

The two coping styles (dominant—subordinate) did not exhibit any statistically significant difference in trunk cortisol whether treated with fluoxetine or not (Figure 2). However, the fluoxetine treated group showed a statistically significant (*P* = 0.004) higher concentration of whole body cortisol in both coping styles than the non-treated group (Figure 2).

### mRNA expression levels between dominant and subordinate not-treated males

**Figure 3:**
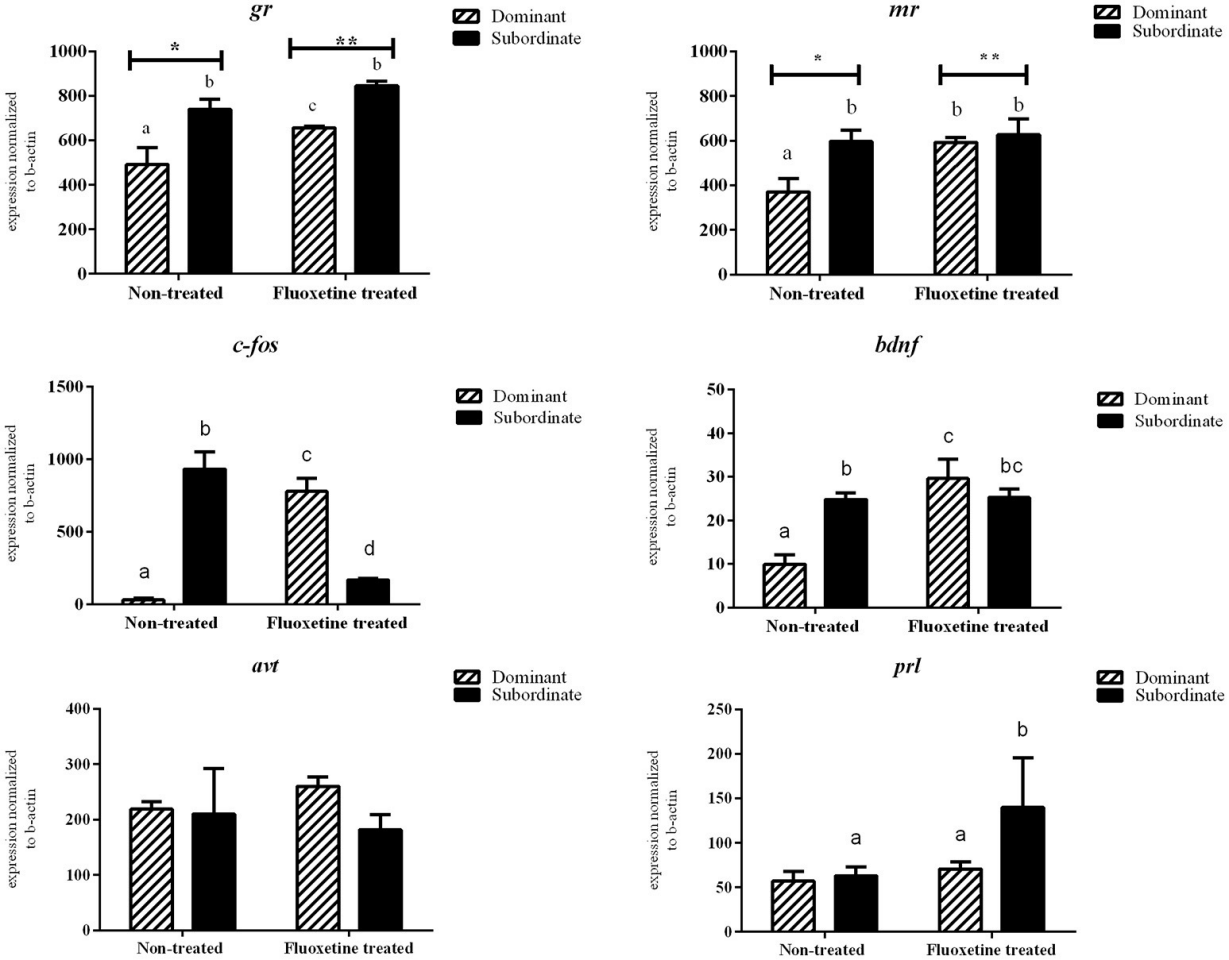
**Relative mRNA expression levels (mean ± S.E.M., *n* = 4 pools of two brains) of *gr*, *mr*, *c-fos*, *bdnf*, *avt*, *prl* in whole brain samples**. Different letters indicate statistically significant differences in all pairwise multiple comparison procedures, while asterisks differences among the two treatments (fluoxetine or non-treated groups).

**Figure 4:**
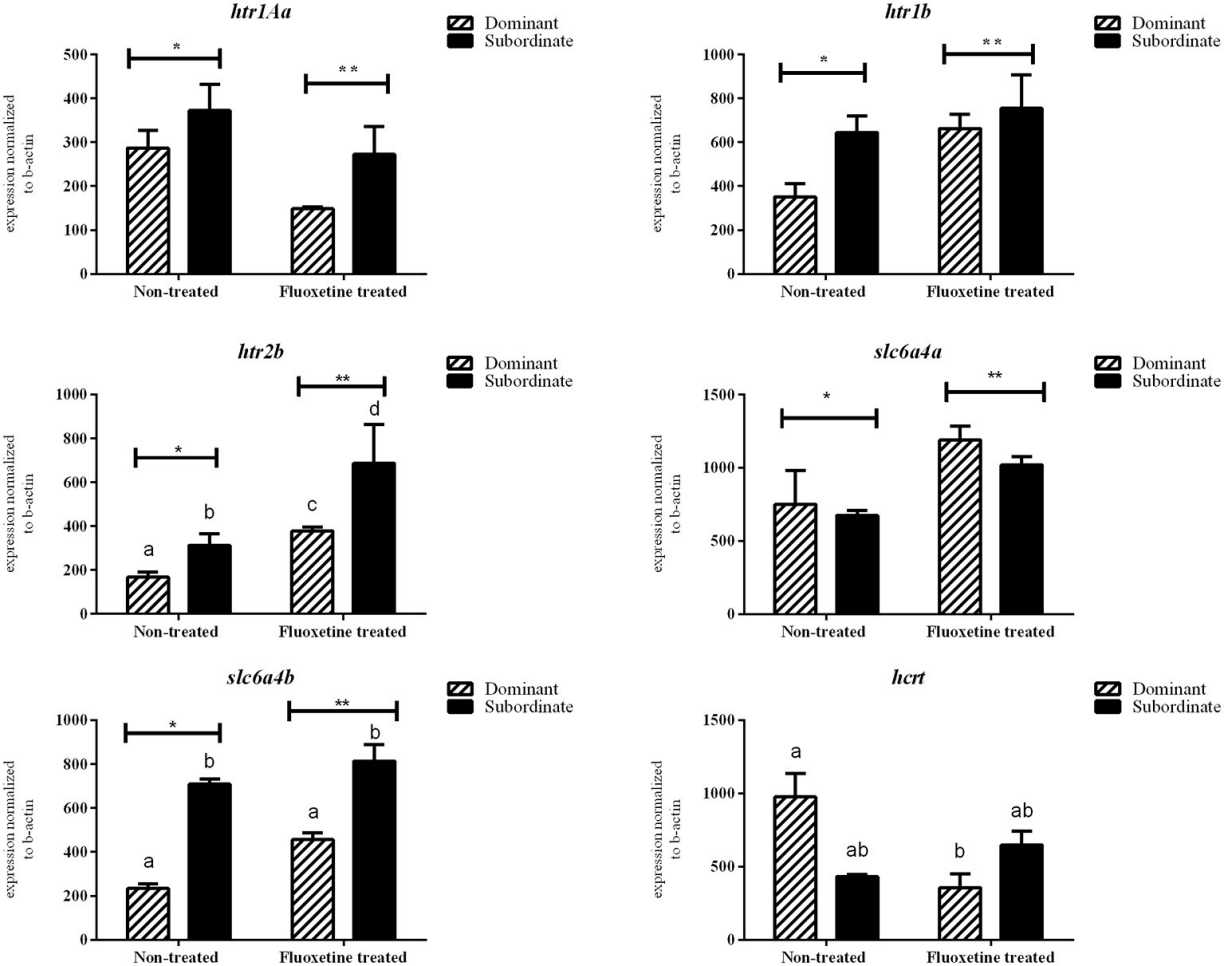
**Relative mRNA expression levels (mean ± S.E.M., *n* = 4 pools of two brains) of *htr1Aa*, *htr1b*, *htr2b*, *slc4a6a*, *slc4a6b, hcrt* whole brain samples**. Different letters indicate statistically significant differences in all pairwise multiple comparison procedures, while asterisks differences among the two treatments (fluoxetine or non-treated groups).

There was no statistically significant difference in the mRNA transcripts of *htr1Aa, htr1B, slc6a4a, avt, hcrt*, and *prl* between dominant (DOM) and subordinate (SUB) non-treated zebrafish (Figures 3, 4). However, non-treated SUB individuals showed a statistically significant up-regulation in *gr* (*P* = 0.011), *mr* (*P* = 0.044), *c-fos* (*P* < 0.001), *bdnf* (*P* = 0.003), *htr2B* (*P* = 0.023), and *slc6a4b* (*P* = 0.007) mRNA expression levels compared to non-treated DOM fish (Figures 3, 4).

### Effect of fluoxetine treatment on mRNA expression levels of the selected genes

Fluoxetine (FLX) treatment resulted in an up-regulation of *htr2b* mRNA transcripts in both DOM and SUB animals. In particular, DOM-FLX-treated and SUB-FLX-treated fish, showed respectively a 2.26-fold (*P* = 0.003) and 2.2-fold (*P* = 0.007) in *htr2b* compared to the respective transcripts of non-treated fish. Brain mRNA expression levels of *gr, mr, c-fos, bdnf*, and *htr1b* showed, respectively, a 1.4- (*P* = 0.04), 1.6- (*P* = 0.04), 25.0- (*P* < 0.001), 3.3- (*P* < 0.001), and 1.89-fold (*P* = 0.04) upregulation only in DOM individuals that were treated with fluoxetine compared to the non-treated DOM. Finally, FLX-treated SUB showed a 1.51-fold (*P* = 0.04) upregulation in *slc6a4a* compared to the non-treated SUB.

Fluoxetine treatment resulted in a 1.93- (*P* = 0.02) and 2.7-fold (*P* = 0.03) down-regulation of *htr1Aa* and *hcrt* in DOM animals compared to the respective non-treated DOM. In addition, exposure to fluoxetine resulted in a 5.6-fold (*P* < 0.001) down-regulation of *c-fos* in SUB compared to non-treated SUB animals. Overall, *c-fos* mRNA expression levels were reversed in dominant and subordinate animals as a result of short-term fluoxetine exposure.

## Discussion

Acute administration of fluoxetine resulted in reduced aggression in dominant males. More specifically, we observed a significant decrease in offensive aggression, as expressed by a significant decrease in the number of attacks of dominant males to subordinates, as well as on the time spent by the dominant chasing the subordinate. Nonetheless, the obtained behavioral data clearly demonstrated that subordinate behavior was also decreased. While prior to treatment the subordinate spent most of its time immobilized on the lower part of the tank, after treatment there was no incident of freezing behavior. As indicated by the behavioral observations, the antidepressant substance did not reverse the hierarchies and, thus hierarchies remained stable post treatment. However, while the hierarchies remained the same, the freezing behavior was not only significantly reduced but some signs of aggressive behavior (attacks) were expressed by some subordinate animals.

The behavioral data are in accordance with published data (Egan et al., 2009), showing an increase in “boldness” following fluoxetine treatment. Similarly, fluoxetine decreased the aggressive behavior of fighting fish, *Betta splendes* (Dzieweczynski and Hebert, 2012; Kohlert et al., 2012) and the territorial aggression in coral reef fish (Perreault et al., 2003). On the contrary, other published data showed that fluoxetine treated zebrafish experienced a reduced number of attacks but this decrease was not statistically significant (Filby et al., 2010). Maximino et al. (2013) reported that fluoxetine administered in zebrafish at the dose of 2.5 mg kg^−1^ had an anxiogenic action in the scototaxis test but anxiolytic in the novel test tank. In another study (Stewart et al., 2011), fish exposed to an acute immersion of the drug at a concentration of 0.1–10 mg L^−1^ had no effect on behavior in the novel tank. This variance may be attributed in the doses used, experimental design or strain differences in drug and behavioral responses. As fluoxetine has been found in surface waters, its effect on the environment and fish behavior has been studied widely. Environmental concentrations of this SSRI impact specific behaviors involved in reproduction, feeding and predator avoidance in certain wild fish species (Weinberger and Klaper, 2014).

We did not identify a statistically significant difference regarding the whole-trunk cortisol concentrations between dominants and subordinates, regardless the treatment. Similarly, Pavlidis et al. (2011) did not find any significant differences between dominant and subordinate individuals. In addition, we observed that acute exposure to a high dose fluoxetine concentration resulted in increased whole-body cortisol in males of both coping styles, compared to the respective non-treated individuals. This observation is contrary with the function of fluoxetine as a common anti-stress drug, and with several publications on zebrafish (Egan et al., 2009; de Abreu et al., 2014) and other species (Rosado et al., 2011; Piwowarska et al., 2012), showing a significant decrease in cortisol concentrations following fluoxetine treatment. However, it has to be noted that the majority of these studies deals with chronic fluoxetine treatment. Several published studies in rodents, show that acute administration of fluoxetine induces a robust secretion of corticosterone or progesterone, while chronic treatment completely blocks the effects of the acute fluoxetine challenge on both corticosterone and progesterone secretion (Purdy et al., 1991; Duncan et al., 1998). In addition, Fuller and Snoddy (1990), reported that the magnitude of the observed increased corticosterone secretion in response to acute fluoxetine challenge is similar to that induced by several physiological stressors. A single injection with citalopram (another selective serotonin reuptake inhibitor) resulted in increased plasma levels of ACTH and corticosterone in rats in a dose dependent manner (Jensen et al., 1999). Similarly, 40 mg of oral citalopram in humans resulted in increased plasma cortisol concentrations (Hawken et al., 2006). It has been proposed, that fluoxetine induces steroid secretion via an action on the brain and/or the pituitary gland, since pretreatment with dexamethasone (a potent synthetic glucocorticoid that suppresses corticosterone or cortisol secretion) completely blocked the fluoxetine-induced increases in corticosterone and progesterone concentrations that have been observed in rats (Duncan et al., 1998).

Our data clearly show that different behavioral syndromes are associated with differences in mRNA transcripts of genes involved in the regulation of the stress response, anxiety, and neural activation. In addition, exposure to the antidepressant drug fluoxetine resulted in significant alterations in expression levels of specific genes, as well as in the behavior of the animals. It is well-accepted, that glucocorticosteroids play a key role in the regulation of physiological, metabolic and behavioral responses to acute and chronic stressors. When an animal is exposed to a challenge or stressor for the first time, it should mobilize all its capacity to respond in a fast and effective manner. This requires the manifestation of various behaviors, including offensive and defensive pattern when the challenge is social. Following fluoxetine exposure a significant up-regulation was observed in *gr* and *mr* transcripts in dominants, indicating that lower brain transcripts of these genes may be associated with the attenuation of defensive aggression. However, a similar up-regulation following fluoxetine exposure was observed in *mr* transcripts only in dominants. It has to be noted that the relationship between serotonin (5-HT) and glucocorticoids is complex (Summers and Winberg, 2006) and depends on context and timing and, in general, chronically high glucocorticoids levels inhibit aggression, but acutely applied corticosteroids do not.

Brain-derived neurotrophic factor is, among else important for the growth, differentiation and maintenance of nerve cells, synaptic plasticity, learning, and long-term memory. Physical exercise is shown to increase BDNF synthesis in the human brain (Denham et al., 2014; Szuhany et al., 2015) and chronic stress increases brain BDNF transcripts in zebrafish (Pavlidis et al., 2015). BDNF-restricted knockout mice exhibited elevated conspecific aggression and social dominance in parallel with increased anxiety and deficits in cognition (Ito et al., 2011). In the present study significantly lower mRNA bdnf expression levels were found in the brain of the non-treated dominant males compared to the subordinate ones. Following fluoxetine treatment there was an increase in bdnf transcripts in the brain of dominants to levels similar to that of the subordinate individuals, supporting the hypothesis that BDNF serve as an anti-aggression molecule. It is known that both BDNF and 5-HT signaling systems regulate the development and plasticity of neural circuits involved in important mood disorders like depression and anxiety. In addition, administration of selective serotonin reuptake inhibitors (SSRIs) resulted in increased bdnf gene expression (Martinowich and Lu, 2008). Therefore, it seems that also in zebrafish, the observed reduced offensive behavior in fluoxetine treated animals, is mediated through increased bdnf expression in the brain of dominant fish.

Fluoxetine treatment completely reversed the pattern of changes in *c-fos* expression levels between dominant and subordinate fish. Non-treated subordinates had almost 30 times higher *c-fos* transcripts that the dominants, while fluoxetine treatment resulted in ~25-fold increase and 5-fold decrease in the brain of dominants and subordinates, respectively. Upregulation of *c-fos* mRNA in a neuron indicates recent activity, thus, expression of this immediate-early gene has been used as an indirect marker of neuronal activity (Krukoff, 1999). Studies in rats have shown that *c-fos* was markedly induced in several brain areas, following fluoxetine administration (Torres et al., 1998; Fraga et al., 2005). In another study in rats, conditioned stress-induced *c-fos* expression in nearly 50 brain regions was observed, while administration of the anxiolytic drug diazepam resulted in dose-related decreases in the frequency of crouching (freezing), as well as in decreased *c-fos* expression (Beck and Fibiger, 1995). Therefore, our results clearly show that in male zebrafish lower or higher neuronal activation in the brain is associated with offensive or defensive aggression, respectively.

The serotonergic system is implicated in several physiological, neuroendocrine and behavioral processes, including stress, anxiety and aggression. The activity of serotonin depends on both the serotonin receptors and transporters; and fluoxetine, as every SSRI, binds to serotonin transporters to block the 5-hydroxytryptamine (5-HT) reuptake in the synaptic cleft allowing 5-HT to exert its effects for a longer duration (Wong et al., 2013). Zebrafish has two serotonin transporter genes; *slc6a4a* and *slc6a4b* (previously serta and sertb; Wang et al., 2006) that mediate serotonin re-uptake (Norton et al., 2008). In our study, we analyzed expression levels of two transporter genes (*slc6a4a, slc6a4b*) and three receptor genes (*htr1B, htr2B, htr1Aa*), with significant differences overall between treatment and coping styles. More specifically, the significant up-regulation observed in *htr1b* in FLX-DOM and in *htr2b* in both FLX-DOM and FLX-SUB, supports published data in other species (Simon and Lu, 2005), showing that activation of the serotonin receptors 1b and 2b is involved in aggressive behavior and indicating that the two receptors are important mediators of offensive and defensive aggression. It has to be noticed that our results are based on whole brain homogenates and an analysis of specific brain regions is needed to elucidate the exact role of those receptors subtypes in coping styles and behavioral syndromes (Takahashi et al., 2011).

Serotonin transporters (*slc6a4a, slc6a4b*) in previous studies did not show any differences following zebrafish exposure to fluoxetine (Wong et al., 2013), without, however, differentiating between coping styles. Dominant individuals, irrespective of treatment, had statistically significant lower expression levels of *slc6a4b* than subordinate individuals, indicating that this difference may be associated with coping strategies in adult male zebrafish.

In conclusion, an acute immersion in a high dose of fluoxetine caused a significant decrease in the aggressive behavior of dominants and an increase in “boldness” of subordinate zebrafish, in association with important differences in molecular modulators implicated in neural function, stress and anxiety (bdnf, c-fos, serotonin transporters, and receptors). Thus, acute fluoxetine treatment may provide a useful neuropharmacological tool to get a better insight into the role of the serotonergic system in social stress and aggressive behavior in adult zebrafish.

## Author contributions

AnT and MP designed the experiment, analyzed data and wrote the manuscript. AnT implemented the experiment and carried out behavioral analysis and cortisol measurements. AlT designed the primer sequences. AlT and AnT performed the mRNA expression studies. AnT prepared Figures 1–4. All authors reviewed the manuscript.

## Funding

The research was partially funded from the European Union Seventh Framework Programme (FP7/2010-2014) under grant agreement no. (265957).

### Conflict of interest statement

The authors declare that the research was conducted in the absence of any commercial or financial relationships that could be construed as a potential conflict of interest.
